# Extraoral Taste Buds on the Paired Fins of Damselfishes

**DOI:** 10.1093/iob/obac035

**Published:** 2022-08-11

**Authors:** Adam R Hardy, Melina E Hale

**Affiliations:** Department of Organismal Biology and Anatomy, The University of Chicago, 1027 E. 57th Street, Chicago, IL 60637, USA; Department of Organismal Biology and Anatomy, The University of Chicago, 1027 E. 57th Street, Chicago, IL 60637, USA

## Abstract

Some fish species have taste buds on the surface of their bodies and fins, as well as in the oral cavity. The extraoral taste system of fish has traditionally been studied in species that inhabit environments and/or employ feeding strategies where vision is limited. Here we examined taste sensation in a new ecological context by investigating the paired fins of damselfish (Pomacentridae), a group of diurnal midwater fishes that inhabit the light-rich waters of coral reefs. Immunohistochemistry demonstrated the presence of taste buds on the paired fins of *Chromis viridis*, including on the distal tips of elongate leading-edge pelvic fin rays, where they are particularly densely packed, suggesting specialization for chemosensation. Similar anatomical results were also recorded from two other species, *Pomacentrus amboinensis* and *Pomacentrus coelestis*. We found that afferent pectoral fin nerves of *C. viridis* responded to a food-derived stimulus. By investigating the extraoral taste system in a new phylogenetic and ecological context, these results show that taste buds on fins are more widespread amongst fish than previously known and are present even in highly visual environments.

## 摘要:(Chinese)

某些鱼种除了在口腔内, 在身体表面以及鱼鳍上也具有味觉受体。在过去对于口腔外味觉感应系统的研究, 传统上是针对那些生活环境或掠食策略受到视觉限制的鱼种。我们在此篇研究当中检视了雀鲷科 (Pomacentridae) 的成对偶鳍, 雀鲷科是一种日行性并生活在光照充足的珊瑚礁的中层水域鱼种, 我们藉由研究雀鲷, 检视了味觉感应在不同生态环境下所扮演的角色。免疫组织化学染色显示了在蓝绿光鳃鱼 (*Chromis viridis*) 的成对偶鳍上有味蕾, 包括了密集聚集在腹鳍前沿远端鳍条上的味蕾, 这可能是为了化学感应而特化的结构。对于另外两种雀鲷科鱼类安邦雀鲷 (*Pomacentrus amboinensis*)以及霓虹雀鲷(*Pomacentrus coelestis*) 的解剖学研究也有相似的结果。我们发现蓝绿光鳃鱼的胸鳍传入神经纤维对食物讯号刺激有反应。通过在新的亲缘与生态系统中研究口腔外味觉系统, 这些结果表明, 鳍上的味蕾在鱼类中比以前已知的更为普遍, 即使在需要高度依赖视觉的环境中也存在。

## Introduction

Taste is fundamental to vertebrate feeding behaviors and is mediated by taste buds, the peripheral sensory organs for gustation. Each taste bud is a pear-shaped structure containing specialized epithelial cells, including taste receptor cells that form a synapse with peripheral sensory neurons (Finger 1997; Reutter and Witt 1999; Hansen et al. 2002; Webb et al. 2019). Unlike most vertebrate groups, in which taste buds are restricted to the oropharyngeal region, fishes can have taste buds all over the surface of the bodies and fins. Investigations of these extraoral taste buds have focused primarily on demersal fishes such as the silurids (Atema 1971; Sakata et al. 2001; Northcutt 2005; Nakamura et al. 2017), cyprinids (Davis and Miller 1967; Gomahr et al. 1992), mullids (McCormick 1993), acipenserids (Kasumyan 1999; 2002; Shamushaki et al. 2011), and gadids (Harvey and Batty 1998, 2002; Kotrschal et al. 1993). These groups tend to be found in vision-limited environments (i.e., living close to the bottom, being nocturnal, and/or inhabiting murky water) and often feed on cryptic or buried prey for which vision is not well-adapted. Electrophysiological recordings from external chemoreceptors, presumably taste buds, have shown that amino acids, natural food extracts, inorganic salts, and various acids are effective stimuli (Bardach and Case 1965; Caprio 1975; Funakoshi et al. 1981; Davenport and Caprio 1982). The distribution and density of extraoral taste buds often reflects their ecology and feeding habits. For example, taste bud density is typically lower in planktivorous and surface-feeding cyprinids than it is in bottom feeders (Davis and Miller 1967; Gomahr et al. 1992).

The paired fins of fishes function in a diversity of behaviors. For those with taste buds, fins can function as short-range chemical detectors and can locate food sources, even, in some species, by means of taste alone (Bardach et al. 1967). Gustatory responses have been recorded from nerve fibers extending through the finger-like pelvic fins of the hake (*Urophycis chuss*) and tomcod (*Microgadus tomcod*) as well as the paired fins of rockling (*Ciliata mustela*), with all species showing sensitivity to amino acids and food extracts (Bardach and Case 1965; Fujiya and Bardach 1966; Peters et al. 1991). Distribution and density of taste buds on fins are known from only a few locations on the paired and median fins among primarily bottom-associated species (Gomahr et al. 1992; ÇInar et al. 2008; Harvey and Batty 1998, 2002). Taste bud density has been shown to be higher along fin margins and lower in more interior fin regions. On a given fin ray, taste buds are distributed largely along the long axis of the fin rays and follow fin ray branching patterns (Nakamura et al. 2017).

Here we investigated the distribution of taste buds on the paired fins of damselfish (family *Pomacentridae*), an ecologically diverse group with at least 422 species (McCord et al. 2021). Damselfish provide the opportunity to investigate the extraoral taste system in an ecological context far removed from those previously studied. The vast majority of damselfishes inhabit the shallow, clear, and light-rich waters of coral reefs, a highly visual environment where the utility of extraoral taste buds on fins is unknown. Furthermore, as primarily diurnal planktivores, damselfishes are known to be visual predators, and some have been shown to possess exceptional visual acuity, including color discrimination, as well as the ability to detect ultraviolet and polarized light (Hawryshyn et al. 2003; Mussi et al. 2005; Siebeck et al. 2008; Cortesi et al. 2020).

We report here on the morphology, spatial distribution, and physiology of taste buds on the paired fins of the blue green chromis (*Chromis viridis*). Found in large aggregations high in the water column above coral heads, *C. viridis* uses their pectoral fins to move through the water while feeding almost exclusively on zooplankton (Coughlin and Strickler 1990; Leray et al. 2019). The paired pelvic fins exhibit an elongated leading edge composed of a small spine and a soft bony ray whose robust distal tips extend well past the margin of the trailing rays. We used antibody staining to map and quantify the full array of taste buds across the pectoral and pelvic fin. To more broadly assess the presence of taste buds among pomacentrids, we examined two species of damselfish from the genus *Pomacentrus* (*P. amboinensis* and *P. coelestis*) that vary in their position on the reef, diet, and gross fin morphology. *Pomacentrus coelestis* and *P. amboinensis* are in different regions of the *Pomacentrus* phylogeny (McCord et al. 2021), while *C. viridis* is in a separate subfamily of Chrominae, which is distant from *Pomacentrus* within the family (McCord et al. 2021; Tang et al. 2021). To confirm the gustatory capability of damselfish fins, we recorded the responses of sensory nerves running along the pectoral fin rays of *C. viridis* to a food-derived taste stimulus. As the first study to investigate taste buds on the fins of a coral reef species, the results presented here suggest that fishes inhabiting a myriad of habitats and environmental conditions utilize taste input from fins.

## Methods

### Animals

Fish were obtained commercially and maintained in separate aquaria as part of a 1200 L saltwater flow-through system at the University of Chicago (Chicago, IL). Blue green chromis (*C. viridis*) are zooplantivores found in large aggregations high in the water column above staghorn coral heads (Coughlin and Strickler 1990; Leray et al. 2019). Neon damselfish (*P. coelestis*) are found near the bottom amongst coral rubble and feed on zooplankton and to a lesser extent on benthic algae (Hobson and Chess 1978; Hamner et al. 1988). Ambon damselfish (*P. amboinensis*) inhabit sandy areas around outcrops and feed mostly on algae, but also consume zooplankton (Sano 1984). Individuals used for experiments were euthanized in a 0.5 g L^–1^ solution of MS-222 (Tricaine methanesulfonate, Sigma-Aldrich, St. Louis, MO) in tank water. All experimental, housing, and euthanasia protocols were approved by the University of Chicago Institutional Animal Care and Use Committee (ACUP Protocol #71589).

### Neuroanatomy of damselfish paired fins

Antibody staining methods were modified from Thorsen and Hale (2007) and Svoboda et al. (2001). The pectoral and pelvic fins from three *C. viridis* (2.6–3.8 cm SL), *P. amboinensis* (3.1–3.6 cm SL), and *P. coelestis* (4.6–5.4 cm SL) were stained and imaged. Fins were preserved in 4% paraformaldehyde in phosphate-buffered saline (PBS) overnight at 4°C. To permeabilize tissues, fins were incubated for 24 h at 4°C in PBS containing 1.0% Triton X-100. Fins were then blocked at room temperature in 10% normal goat serum (NGS) in PBS containing 0.1% Tween-20 and 0.5% Triton X-100 for 1 h.

Fins were incubated at 4°C in a blocking solution with both primary antibodies. Nerves were stained using a mouse monoclonal anti-acetylated tubulin antibody (aat, Sigma-Aldrich) at a final concentration of 1:250. Receptor cells within taste buds were stained using a rabbit monoclonal antibody, CR 7697, directed against calretinin (Swant Antibodies, Bellinzona, Switzerland) at a final concentration of 1:1000. Previous studies have used calretinin, a calcium binding protein, to detect taste buds in a variety of fish species (Díaz-Regueira et al. 2005; Northcutt 2005; Germanà et al. 2007; Varatharasan et al. 2009; Nakamura et al. 2017). After 48 h, fins were rinsed three times for 30 min each with PBS and then incubated at 4°C in blocking solution containing both secondary antibodies goat anti-mouse IgG (H + L) cross-adsorbed, Alexa Fluor 546 (Thermo Fisher Scientific, Waltham, MA, USA) and a goat anti-rabbit IgG (H + L) cross-adsorbed, Alexa Fluor 647 (Thermo Fisher Scientific) at a final concentration of 1:250. Fins were removed from secondary antibodies after 1 to 2 days, rinsed three times for 30 min each with PBS, and stored in PBS @ 4°C until they were imaged.

### Taste bud density and distribution analysis

The pectoral and pelvic fins of three individuals per species were imaged using a Caliber I.D. RS-G4 confocal microscope (Rochester, NY). The Z-series stacks of three µm thickness were taken of the fin rays and associated fin membrane. The location and number of taste buds were determined using the “spot” detection feature in Bitplane Imaris software v. 9.0.1 (Andor Technology PLC, Belfast, N. Ireland). Counts reported here are conservative estimates that only include clearly visible and well-defined taste buds. Our analysis missed some taste buds at the distalmost tips of fin rays where strong anti-calretinin activity and the high density of taste buds made it impossible to resolve the boundaries of all of the taste buds, which resulted in undercounting.

**Fig. 1:**
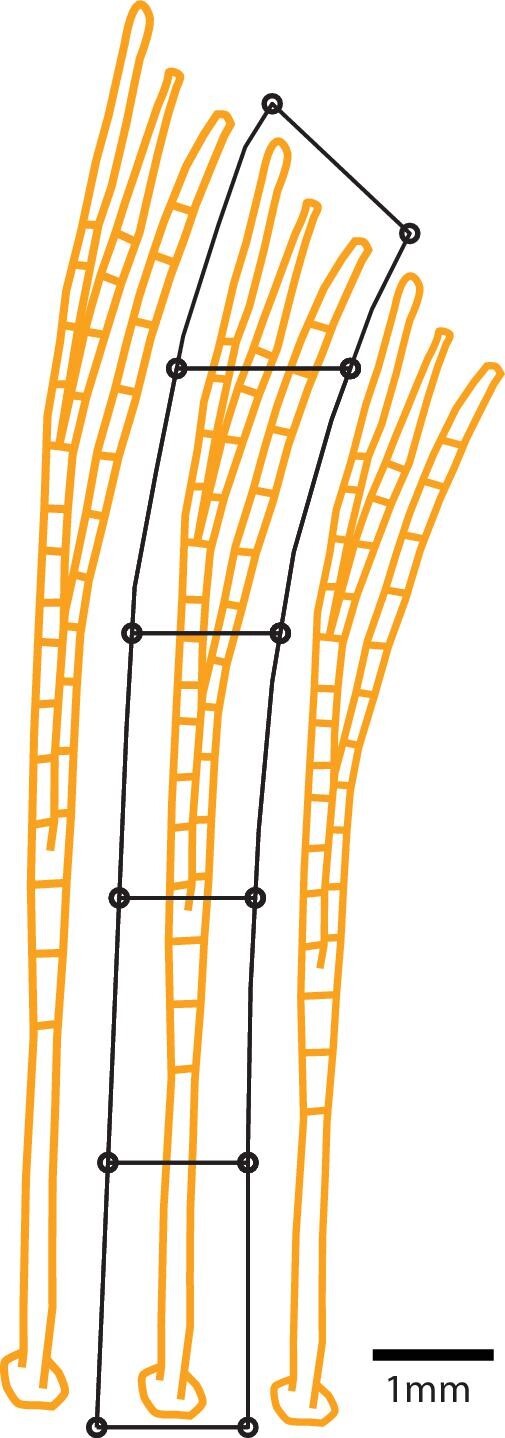
Methodology to determine the spatial distribution of extraoral taste buds. Taste buds were identified using the “spot” detection feature in Bitplane Imaris software v. 9.0.1 (Andor Technology PLC, Belfast, N. Ireland). Density estimates were then calculated within 5 contiguous ROI's along the proximodistal axis of a given fin ray using a custom MATLAB script. Each ROI spanned 20% of the fin ray length and extended laterally to a point equidistant to the adjacentmost ray.

The density and distribution of taste buds were quantified within five contiguous regions of interest (ROI) along the proximodistal axis of each fin ray using a custom MATLAB script (Mathworks, Natick, MA). As fin rays were each of a different length, each ROI spanned 20% of the fin ray length and extended laterally to a point equidistant to the adjacent ray (Fig. 1). Taste buds within each ROI were counted and a density measurement (taste buds/mm^2^) calculated. On the pectoral and pelvic fin, fin rays # 1 and 2 were combined into a single ROI to capture the full extent of leading-edge innervation. Similarly, the last two rays of the pectoral fin were combined to capture the full extent of innervation on the trailing edge of the fin. In addition to these leading and trailing edges as discussed above, we selected pectoral fin rays #6, 9, 11, and 15 of *C. viridis*, #5, 8, 10, and 13 of *P. amboinensis*, and #5, 8, 10, and 14 of *P. coelestis* as well as pelvic fin ray #4 and 6 for taste bud density calculations.

### Physiological responses

We recorded from afferent nerves within the pectoral fin of *C. viridis* in response to chemical stimulation. These sensory fibers within the pectoral fin are believed to be of cranial and spinal origin (Kotrschal et al. 1993; Kiyohara and Caprio 1996; Thorsen and Hale 2007; Ma et al. 2010). After euthanizing the fish in MS-222 (0.5 g/L), the pectoral fin and associated musculature were excised from the body and placed in a Petri dish (100 mm × 15 mm) filled with extracellular solution that contained (in mM) 134 NaCl, 2.9 KCl, 1.2 MgCl_2_, 2.1 CaCl_2_, 10 HEPES buffer, and 10 glucose, adjusted to pH 7.8 with NaOH. Extracellular solution, made according to the methods found in Masino and Fetcho (2005), is a physiological saline solution commonly used in fish physiology experiments. Electrophysiology methods followed Williams IV et al. (2013). Briefly, we recorded multiunit physiological responses from nerves entering the right pectoral fin of three individuals using glass suction electrodes (GC150F-7.5 1.5 mm OD, 0.86 mm ID, Harvard Apparatus, Holliston, MA). Nerve recordings were taken from the median side (facing the body) of the fin. In the physiology preparation, the median side was oriented up to provide the best access to the sensory afferents.

The chemical stimulus used in these experiments was prepared prior to each experiment by thawing an approximately one square inch of frozen brine shrimp (Sally's Frozen Brine Shrimp; San Francisco Bay Brand) in 50 mL of extracellular solution. The mean number of brine shrimp calculated from three representative solutions was 910 (range: 853−998). The amino acid composition of brine shrimp (*Artemia spp.*) includes high levels of alanine, arginine, aspartic acid, glutamic acid, and proline, which have been shown to be effective at generating a taste response in other fish species (Gallagher and Brown 1975; Corazza and Saylor 1983; Morais 2017). Once the brine shrimp had fully thawed, the solution was passed through Whatman grade 1 filter paper and then further filtered using a 0.2-μm Corning syringe filter (Corning Inc, Corning, NY). The filtrate was stored at 4°C and used within 48 h. Experiments began with assessing the responsiveness of afferents within the pectoral fin rays to the application of the chemical stimulus. Once a positive response was localized to a particular fin ray region, the extracellular solution in the petri dish was replaced with a fresh solution using a large pipette. As a control, we applied extracellular solution to test for the possible effects of fin ray displacement due to water movement produced by the application of the stimulus.

Stimuli and control solutions were delivered at room temperature by a picospritzer unit (Picospritzer III, Parker-Hannafin, Pine Brook, NJ, United States) using separate 1 mL tuberculin syringes (Henke Sass Wolf, Tuttlingen, Germany), both equipped with a 0.2‐μm Corning syringe filter (Corning Inc., Corning, NY) and a 27-gauge × 1/2 needle (BD Precision Glide, Franklin Lakes, NJ). These syringes were mounted in parallel to a motorized manipulator (Siskiyou MX7600R) with the needle tips positioned at a ∼45° angle above the fin ray surface. Each trial consisted of the application of the chemical stimulus followed by the control and the subsequent application of the chemical stimulus. In order to prevent effects due to adaptation, we maintained inter-stimulus intervals of at least 1 min. The pulse pressure and duration of the picospritzer unit were set to maintain a total injected volume of ∼10 uL. Video of the stimuli was recorded using a Fastcam APX RS camera (Photron, San Diego, CA).

Data were analyzed in MATLAB 2017a (Mathworks, Natick, MA). To identify and sort individual units from our extracellular recordings, we used a modified version of the spike sorting algorithm, Wave_clus (Quiroga et al., 2004). Statistical analyses of the mechanical stimulation data were performed using JMP software (SAS, Cary, NC). We applied a firing rate threshold (mean + 4* standard deviation [SD]) to identify spikes associated with the burst of stimulus-evoked activity. Each afferent’s activity in response to the control solution was characterized between the time period associated with the first and last spike of the stimulus evoked burst of activity identified in the previous stimulus application.

## Results

### Taste bud identification and morphology

**Fig. 2:**
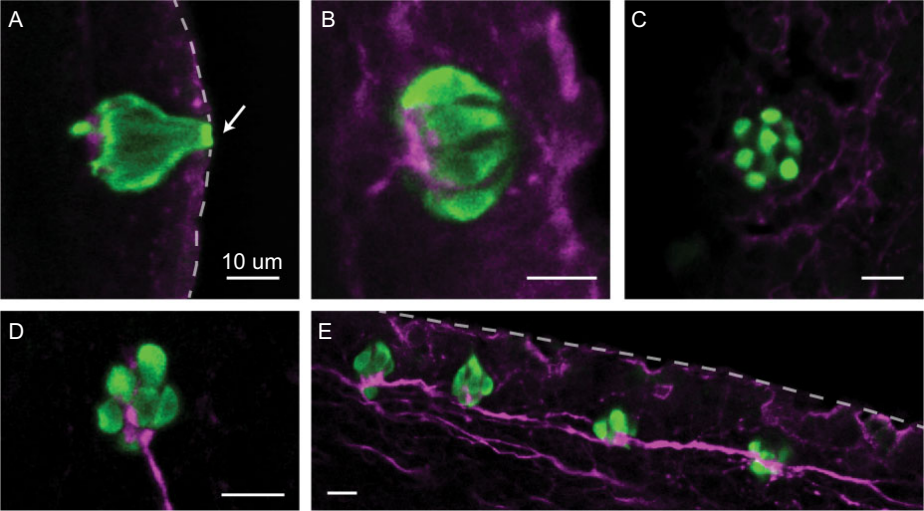
Morphology of extraoral taste buds on the paired fins of *C. viridis*. (**A**) Located at or in close proximity to the epidermal surface, taste buds (anti‐calretinin, green) are small (∼10–25 μm diameter) pearshaped sensory structures that extend to the external surface via a 3–5 μm diameter pore (arrow) at their apical end. (**B**) Taste buds are composite structures composed of multiple cell types including the elongated receptor cells shown here. (**C**) The number of these calretinin-positive cells within each taste bud varies from ∼6 to 10. (**D**) Nerve fibers (AAT, magenta) terminate in an expanded nerve plexus at the base of each taste bud. (**E**) We often observed nerve fibers innervating multiple taste buds, suggesting that along a given fin ray, taste buds may operate as a functional unit. Dashed lines mark the edge of the fin. Scale bars: 10 um.

Immunolabeling identified calretinin-positive clusters of cells on both the pectoral and pelvic fins of all three damselfish species examined here. The morphological characteristics of these newly identified bulbous endings in damselfish are consistent with those of taste buds identified previously in studies of other species. Located at or in close proximity to the epidermal surface, damselfish taste buds are small (∼10–25 μm diameter) and protrude to the external surface via a pore of ∼4 μm diameter (Fig. 2A). Each taste bud is a composite structure consisting of ∼6–10 elongated pear or onion-shaped receptor cells (Fig. 2B and 2C). Immunolabeling with a general neuronal marker revealed an organized network of sensory fibers that extend distally within each ray, following fin ray branching patterns. A subset of them terminated in expanded tufts at the base of each taste bud (Fig. 2D). In some locations, nerve fibers innervated multiple taste buds (Fig. 2E). While not shown here, taste buds were found on the dorsal, anal, and caudal fins, further suggesting the importance of chemosensory input from damselfish fins.

### Taste bud distribution and density

**Fig. 3:**
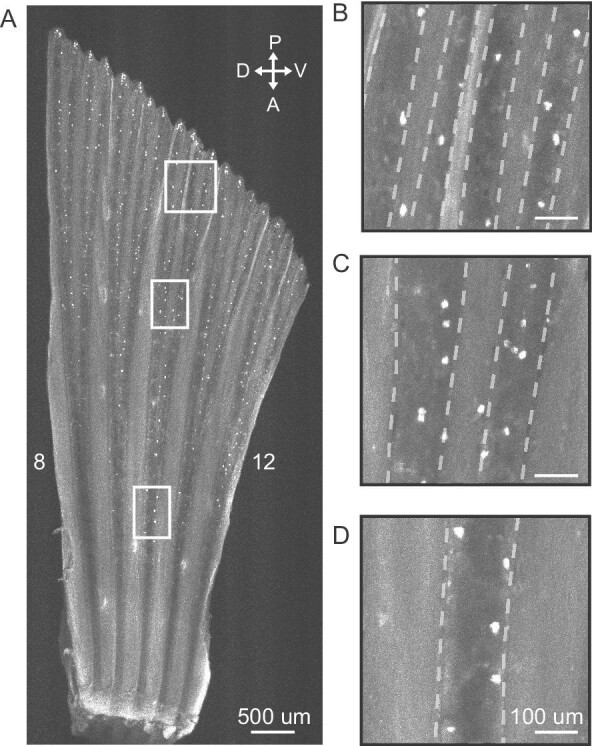
Taste buds on the pectoral fin rays of *C. viridis*. (**A**) Greyscale image of calretinin fluorescence showing taste buds across five middle (#8–12) pectoral fin rays. Boxes show regions enlarged in (**B**)–(**D**). Taste buds are distributed parallel to the proximodistal axis of a fin ray. (**B**)–(**D**) We found that taste buds were located on or in very close proximity to the fin rays themselves. Taste buds were rarely observed within the inter-ray membrane (dashed lines mark the edges of the fin rays). As such, taste buds in proximal, nonbranched regions of a given fin ray are linearly arranged in a column. However, as fin rays branch distally, this single row of taste buds observed proximally branch to follow fin ray branching patterns. Taste bud density was highest at the distalmost tips of fin rays. Scale bars: 500 μm in A; 100 um in B–D.

We describe the distribution of taste buds across the paired fins of *C. viridis*, which are similar to those of *P. coelestis* and *P. amboinensis*. Taste buds are distributed largely parallel to the long axis of the fin rays and follow existing fin ray branching patterns (Fig. 3). Taste buds were located on or in very close proximity to the fin rays themselves, with very few receptors distributed within the inter-ray membrane. On leading and trailing edge fin rays, taste buds are localized to the exterior edge of the rays, whereas on central fin rays (i.e., fin rays #5–13), taste buds could be found throughout a given ray with no apparent localization to a particular edge.

**Fig. 4:**
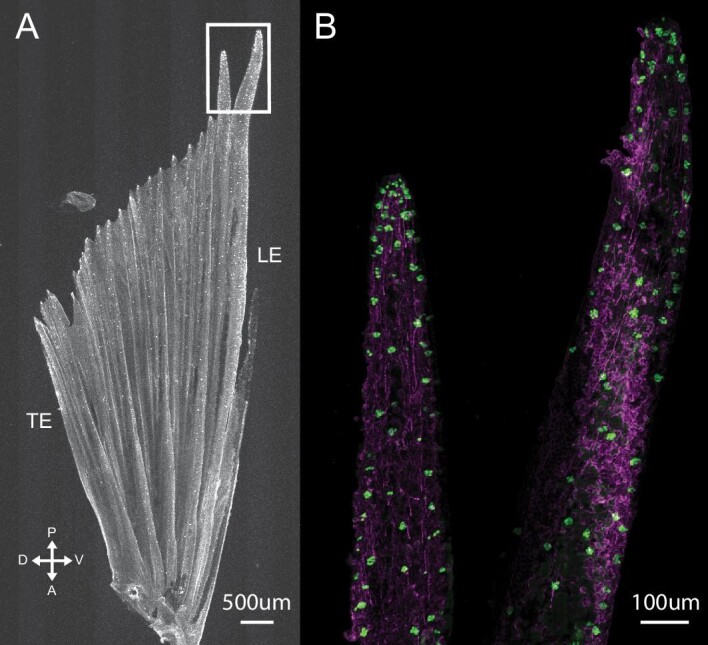
Taste buds on the pelvic fin of *C. viridis*. (**A**) Greyscale image of calretinin fluorescence showing taste buds (white dots) across the pelvic fin. Taste buds were most prominent along the leading edge, with a significant concentration found toward the distal tips of pelvic fin ray #2. White box shows a region enlarged in (**B**). (**B**) Immunostained distal tips of pelvic fin ray #2 showing nerves (AAT, magenta) and taste buds (anti‐calretinin, green). The distal tips of this fin ray are robust and unbranched, and both extensions of the ray extend well past the margin of the trailing rays. We found that these areas were densely populated with taste buds compared to the rest of the pelvic fin. Scale bars: 500 μm in A; 100 μm in B.

On the pectoral fin, *C. viridis* showed a mean taste bud density (taste buds/mm^2^) of 40.65 ± 20.17 (mean ± SD). The ROI with the highest densities was found along the margins of the fin, including the leading and trailing edges (Fig. 6). Beyond these areas, a prominent proximodistal gradient existed along a given fin ray with few taste buds located proximally and much higher densities distally. Taste bud density was lowest in the proximal regions of central fin rays (i.e., fin rays #5–13). On the pelvic fin, the mean taste bud density was 48.44 ± 20.56 (mean ± SD). Taste buds were most prominent along the margins, with a significant concentration found on the leading edge (Figs. 4 and 7). As noted previously, the first soft ray of the pelvic fin (fin ray #2) extends well past the distal end of the rest of the fin. The distalmost region of this elongated ray in *C. viridis* was heavily innervated with a mean density of 192 taste buds/mm^2^, suggesting the importance of the ray for chemical detection.

**Fig. 5:**
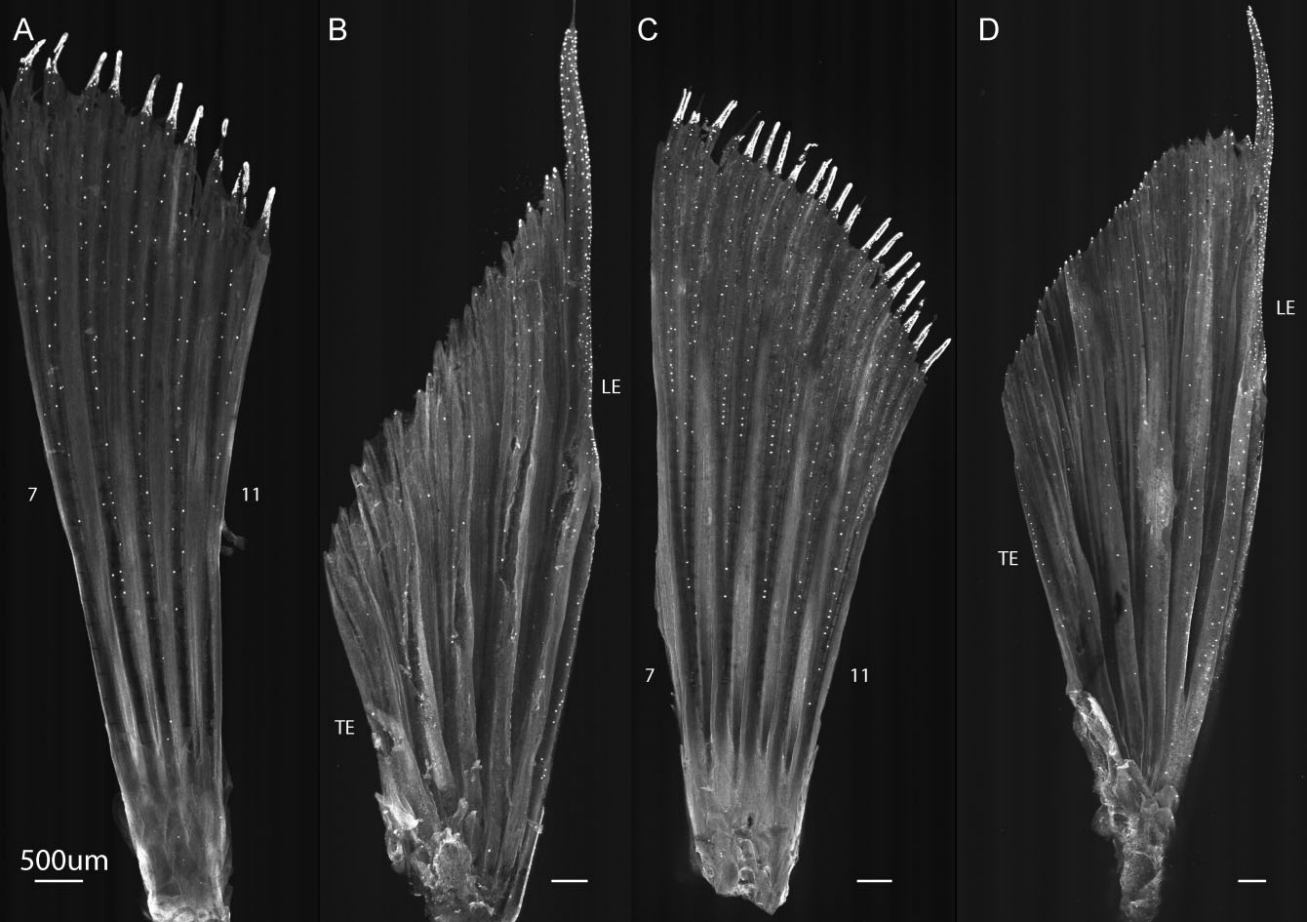
Taste buds on the paired fins of two other distantly related species of damselfish. Greyscale image of calretinin fluorescence showing the distribution of taste buds across pectoral fin rays # 7–11 (**A**), (**C**) and the pelvic fin (**B**), (**D**) of the ambon damselfish (A, B; *Pomacentrus amboinensis*) and the neon damselfish (C, D; *P. coelestis*). (**A**), (**C**) Taste buds on the pectoral fin exhibit distribution patterns similar to those observed in *C. viridis*. Taste buds are distributed largely parallel to the long axis of the fin rays, follow fin ray branching patterns, and are absent from the inter-ray membrane. The distal tips of these rays appear densely populated, but the high florescence intensity in these regions makes resolving individual taste buds impossible. (**B**), (**D**) The pelvic fin of *P. amboinensis* and *P. coelestis* exhibits numerous taste buds along the leading edge, with significant concentrations found toward the distal tips of pelvic fin ray #2. Compared to *C. viridis*, however, only the leading-edge bifurcation of this ray, distal to the first branchpoint, is robust and extends past the margins of the rays. The trailing edge bifurcation is much shorter and retains the classical morphology of more typical soft bony rays. Scale bars: 500 μm.

**Fig. 6:**
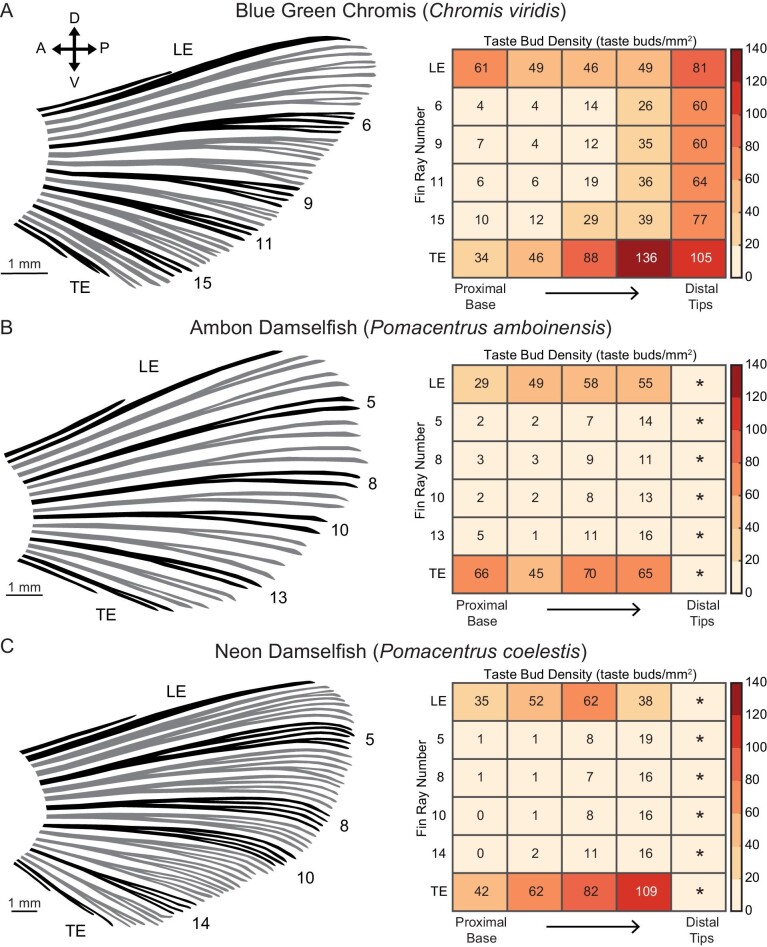
Damselfish pectoral fin morphology and taste bud distribution. Left: Pectoral fin from (**A**) *C. viridis*, (**B**) *P. amboinensis*, and (**C**) *P. coelestis*. Fin rays selected for taste bud analysis are colored black and labeled. Right: Heatmaps show the density of taste buds along the fin rays of interest. Each row shows data collected from a given fin ray. Each cell shows data collected from a given ROI. As fin rays were each of a different length, each ROI spanned 20% of the fin ray length and extended laterally to a point equidistant to the adjacentmost ray. The mean (*n* = 3 individuals) taste bud density (taste buds/mm^2^) for each ROI is marked numerically and is also represented by color (dark red = higher density; light orange = lower density), as indicated in the key. ROI marked with an asterisk were not counted despite clearly being heavily populated with taste buds as the fluorescence intensity made accurate counts impossible. We find that taste buds on damselfish pectoral fins are densely packed on the margins (i.e., edges) of the fin with few taste buds located centrally.

**Fig. 7:**
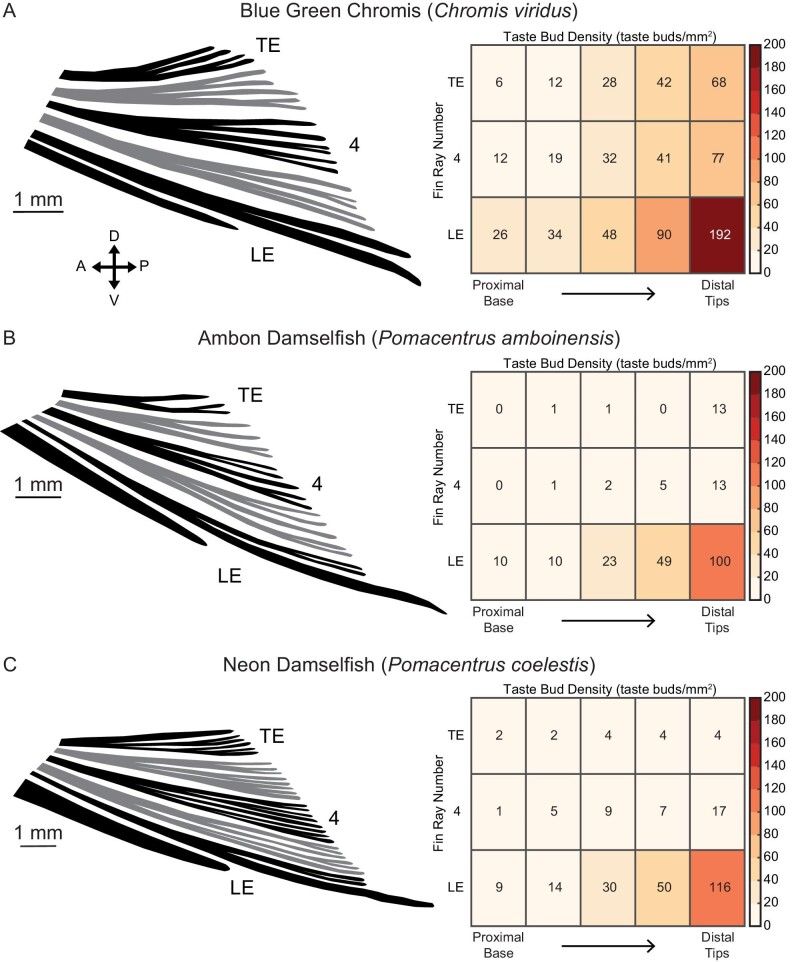
Damselfish pelvic fin morphology and taste bud distribution. Left: Pelvic fin from (**A**) *C. viridis*, (**B**) *P. amboinensis*, and (**C**) *P. coelestis*. Fin rays selected for taste bud analysis are colored black and labeled. Right: Heatmaps show the density of taste buds along the fin rays of interest and are structured as in Fig. 6. Taste buds were most prominent along the margins, with a significant concentration found on the leading edge. The first soft ray of the pelvic fin (fin ray #2) extends posteriorly well past the margin of the rest of the fin. We found that the distalmost ROI along these elongated rays in *C. viridis* was densely populated with a mean density of 192 taste buds/mm^2^, suggesting the importance of this region for chemical detection. This value is approximately double that of comparable fin regions found in *P. coelestis* and *P. amboinensis*, which have only a single robust distal extension of the fin ray. Compared to the pectoral fin, we note a large decrease in taste bud abundance from the leading to trailing edge.

The paired fins of *P. amboinensis* and *P. coelestis* are also densely populated with taste buds, suggesting that taste buds on fins are generalized in damselfish (Figs. 5–7). Densities on the leading and trailing edges of the pectoral fin were fairly similar among the three species examined here. Comparisons of densities between the distal tips of central fin rays for *P. amboinensis* and *P. coelestis* was not possible as the very high florescence intensity in these areas made resolving individual taste buds impossible. On the pelvic fin, leading-edge fin rays (fin rays #1 and 2) are heavily innervated along their lengths compared to the rest of the fin (Fig. 7). The density of taste buds in the distalmost region of pelvic fin ray #2 in *C. viridis* (192 taste buds per mm^2^) is approximately double that of comparable regions found in *P. coelestis* (116 taste buds per mm^2^) and *P. amboinensis* (100 taste buds per mm^2^), which both exhibited only a single robust distal extension of pelvic fin ray #2 (Figs. 5 and 7).

### Physiological response

**Fig. 8:**
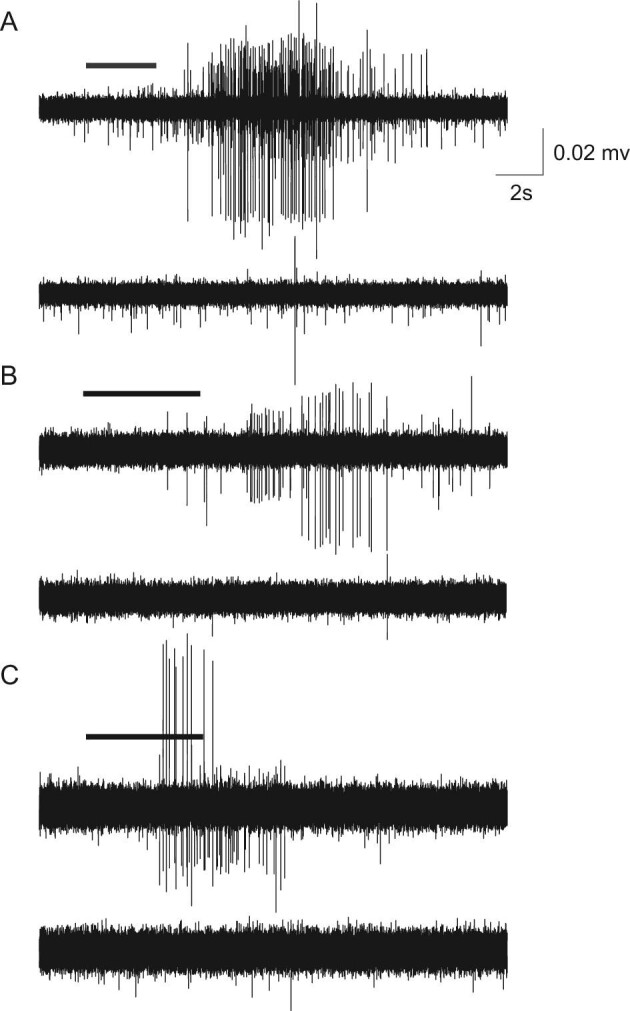
Physiological response of *C. viridis* pectoral fin ray afferents to chemical stimulation. (**A**)–(**C**) Extracellular multi-unit recordings from three separate fish showing the representative response to the stimulus (food extract; top trace) and to the subsequent application of the control (extracellular solution; bottom trace). The duration of the stimulus and control applications is marked by the horizontal black bar. Units responsive to the stimulus exhibited a burst-like response that continued several seconds before returning to baseline. When averaged across afferents (*n* = 19 afferents among three individuals), the spike number and spike rate (spikes/s) associated with the stimulus evoked activity were 40.02 ± 29.67 and 5.66 ± 2.85 (mean ± SD), respectively. Activity in response to the subsequent application of a control solution was significantly reduced (F1,7 = 14.88, *P* = 0.0048) or for the majority of recorded afferents (*n* = 15) entirely absent. Scale bar: *x* = 2 s, *y* = 0.02 mV.

From our multi-unit recordings of *C. viridis* pectoral fin ray nerves, we identified afferents (*n* = 19) from three individuals that exhibited spiking in response to a food-derived stimulus (Fig. 8). Spontaneous activity of these fibers was relatively low, and as such, the burst of stimulus-evoked activity was clearly evident. When averaged across afferents, the spike number and spike rate (spikes/s) associated with this activity were 40.02 ± 29.67 and 5.66 ± 2.85 (mean ± SD), respectively. Once stimulated, afferents continued to fire for several seconds before returning to baseline. Variation in the duration of stimulus evoked activity ranged from 2.26 to 28.03 s with a mean duration of 7.69 s when averaged across trials. Stimulus evoked activity was typically delayed by several seconds relative to the onset of chemical stimulus as time was required for the stimulus once injected into the experimental dish to contact the receptive field of the recorded taste fiber. Relative to the stimulus evoked response, the response to the control solution as measured by spike rate (spikes/s) was significantly reduced (F_1,7_ = 14.88; *P* = 0.0048) and for the majority of these chemosensitive afferents (*n* = 15) it was absent. We measured the stimulus evoked response before and after the application of the control solution. While variation exists amongst afferents, no significant difference was found when averaged across fibers in the number of elicited spikes (F_1,36_ = 0.02; *P* = 0.8850), the spike rate (F_1,36_ = 0.31; *P* = 0.5793), or the duration of stimulus evoked activity (F_1,36_ = 0.03; *P* = 0.8606).

## Discussion

From these results, we conclude that (1) the paired fins of damselfish are densely populated with taste buds, (2) taste bud distributions on the paired fins are well positioned to sense taste-related stimuli at a distance from the body, and (3) sensory input via fins in response to a food-derived chemical stimulus is more common amongst fishes than previously thought.

Fish taste buds are pear or onion-shaped epithelial sensory structures consisting of multiple cell types that include gustatory receptor cells, support cells, and basal cells. While morphological variation exists among species (Kapoor et al. 1976; Reutter and Witt 1999; Reutter et al. 2000), both gustatory receptor and support cells have an elongated shape with their apical ends protruding into the oral cavity or external environment via a small pore. Here, we found that the elongated cells of taste buds across the paired fins of damselfish exhibited strong immunoreactivity to calretinin, a reliable marker of taste buds in a variety of other fish species (Díaz-Regueira et al. 2005; Northcutt 2005; Germanà et al. 2007; Varatharasan et al. 2009; Nakamura et al. 2017) (Fig. 2). Pore diameter (4–5 μm) as well as the number of receptor cells (6–10) within each taste bud are consistent with findings from other species (Jakubowski and Whitear 1990). Labeling of nerves by anti-acetylated tubulin antibody showed that each taste bud is innervated at its base by a network of fibers, but future work will be needed to identify whether nerve fibers in the fins of damselfishes innervate multiple taste buds, thus forming clusters of connected receptors.

The abundance of taste buds on the paired fins of *C. viridis*, together with observations from two damselfish species from different subfamily, indicates that these regions provide considerable chemosensory input throughout the family. As the aquatic environment is rich in dissolved compounds, taste buds on fins provide surface area for chemical detection and extend the sampling area along a fish's length. In addition to responding when in physical contact with potential food items, we argue that taste buds on damselfish fins may also facilitate taste at a distance from the food item through chemicals in the surrounding water. In this study, we found the highest taste bud densities along the fin margins, such as the leading and distal edge, and much lower densities in more proximal fin regions. As damselfish utilize their pectoral fins as the primary propulsors during swimming (Gerstner 1999; Hale et al. 2006; Aguilar-Medrano et al. 2013), this arrangement may be positioning taste buds in regions of the fin most likely to encounter oncoming chemical stimuli. Furthermore, movement of the fins increases the amount of fluid contacting the taste buds, potentially augmenting chemosensation. It has also been hypothesized that this distribution takes advantage of the thinner hydrodynamic boundary layer over the edges of the fin, which may serve to enhance the likelihood of chemical stimuli contacting a given taste bud (Harvey and Batty 1998, 2002). The precise nature of how damselfish pelvic fins function and interact with the surrounding fluid during behavior is unknown, but the abundance of pelvic fin taste buds, particularly along the distal tip extensions of the leading-edge, suggests specialization for chemosensation. Positioned below the body, these pelvic fin extensions significantly extend the sampling area for chemical detection around the body. Future behavioral work that investigates how the pelvic fins are positioned during feeding behaviors will further tease apart the function underpinning the distribution of this sensory anatomy.

While extraoral taste bud density can generally be predicted from a species proclivity to a benthic lifestyle, the data reported here suggest that other ecological and behavioral considerations must be considered. Harvey and Batty (2002) report quantitative data on the abundance of taste buds across the paired fins of several cod-like fish (Gadidae). Densities along the pectoral fin leading edge were typically less than 100/mm^2^, but spot densities of up to 700/mm^2^ were recorded from the more benthic species, and mean densities across the first two pelvic fin rays ranged from 42–398/mm^2^ among species. Gomahr et al. (1992) investigated ten cyprinid species and found mean taste bud densities on the pectoral and pelvic fins of 150 and 132/mm^2^ respectively, with the more benthic fishes typically exhibiting the most taste buds. A notable exception was the Eurasian minnow (*Phoxinus phoxinus*), which exhibited relatively high densities of taste buds across the body and fins despite being found in mid-water in clear creeks and lakes. Similarly, we found that while most regions of the damselfish pectoral fin have fewer than 50 taste buds per mm^2^, densities at the distal tips of leading-edge pelvic fin rays (192 taste buds per mm^2^ for *C. viridis*) are comparable with the fins and even barbels of many species. For example, the barbels of catfish and goatfish probe the substrate in search of food with taste bud densities reaching over 200/mm^2^ at their distal tips (Sakata et al. 2001; Kiyohara et al. 2002). The higher density of taste buds relative to the rest of the paired fins and comparable densities to that of barbels, classically thought of as highly specialized structures for taste sensation, suggest that the modified extensions of the pelvic fin rays may serve a similar function. Taken together, it is likely that body and fin regions from fishes inhabiting a diversity of habitats and environmental conditions possess taste bud at densities previously thought confined only to benthic fishes and structures, such as barbels, which are specializations for taste.

Damselfish species vary in diet and have been classified as herbivores, planktivores, or omnivores that consume both filamentous algae and small animal prey. Of the three damselfish species examined here, we found that *C. viridis* had the highest density of taste buds across the paired fins and hypothesize that this may be a specialization for feeding almost exclusively on zooplankton. *Chromis viridis* forms large stationary aggregations high in the water column while foraging and relies on the current to deliver them planktonic foods. While interpretations of the relationship between sensory morphology and ecology are limited here due to low species sampling, we hypothesize that the increased density of cutaneous taste buds observed in this species better facilitates the detection and localization of upstream food-related chemical cues in the water column. In contrast, *P. coelestis* and *P. amboinensis*, which have lower taste bud densities, are found close to the bottom. As omnivores, their diet includes benthic algae, which may function to lower the demands for gustatory input via fins. The abundance and spatial distribution of taste buds in herbivorous damselfish that feed exclusively on algae would provide useful information on the utility and demands of extraoral taste buds across the range of damselfish diets.

While oral taste buds provide sensory input that has obvious implications during feeding, extraoral taste buds may function in other behavioral contexts. Damselfish are known to utilize chemical alarm cues elicited through mechanical damage from the skin of conspecifics as well as diet cues released upon defecation by a predator to assess the risk of predation (Lönnstedt and McCormick 2011; Ferrari et al. 2017; McCormick et al. 2019). Chemical cues are also known to influence orientation and settlement behaviors among fishes (Atema et al. 2002; Lecchini et al. 2005; Døving et al. 2006; Hu et al. 2019). It has largely been assumed that these types of chemical cues are detected solely by the olfactory systems of fishes, but the chemical composition of these cues is still largely unknown (Ferrari et al. 2010; Mitchell et al. 2017). Furthermore, the olfactory and oral gustatory systems of fishes, while anatomically distinct, are known to detect similar types of chemical stimuli (i.e., amino acids and bile salts) at comparable concentrations (Caprio 1977; Hara 1994). Understanding the types and concentrations of chemical stimuli needed to elicit a response will be critical to understanding the role of taste buds on damselfish fins during both feeding and non-feeding behaviors and to facilitating broader comparisons for how fishes of varying ecology are adapted to detect the abundant chemical stimuli in the aquatic environment.

As one of a few studies to record the electrophysiological response of chemical sensation (potentially from extraoral taste buds) on fins (Bardach and Case 1965; Fujiya and Bardach 1966; Peters et al. 1991), the data reported here further illustrate the need to consider chemoreception in fish fin function. Similar to the chemical responses from the barbels and flank skin of catfish (Caprio 1975; Davenport and Caprio 1982; Marui et al. 1983), we observed a strong burst-like response to a food-derived stimulus from fibers within the pectoral fin. The burst lasted several seconds before returning to the baseline (Fig. 8). A major limitation to interpreting the form and function of fin ray sensory systems is the difficulty of explicitly matching the neural activity to a given receptor or receptor type. Similar to previous electrophysiological studies reporting on extraoral taste buds, we cannot exclude the possibility that these recorded responses are from free nerve endings or solitary chemosensory cells. Based on the morphology and abundance of taste buds across the paired fins of the damselfish *C. viridis*, it is likely that at least a subset of the responses reported here are from extraoral taste buds. Future investigations simultaneously integrating confocal imaging and single-cell electrophysiological techniques with ethologically relevant manipulations of the fins would facilitate the one-to-one mapping of sensory morphology and function necessary to confirm the response of a given receptor. As the roles of extraoral taste buds become clearer, comparison to oral taste buds will also be important. Given the hypothesized need to facilitate taste at a distance, extraoral taste buds on fins may be more sensitive and respond to a broader range of chemical stimuli than those in or near the oral cavity.

We show that cutaneous taste buds are more widespread and likely serve more purposes among fishes than previously understood. The discovery of a well-developed extraoral taste system in an ecological context classically not thought to necessitate sensory input from extraoral taste buds suggests that fishes inhabiting a myriad of habitats and environmental conditions likely utilize chemosensory input from fins. While damselfish, like many other diurnal fishes, are thought to rely on vision during feeding, sensory input from taste buds on their paired fins may complement the input of other sensory systems to maximize efficiency during food searching behaviors. Future investigations should take a comparative approach to understand the full extent of taste buds across the diversity of fishes as well as the ecological factors that influence the placement and number of these receptors on particular regions of the body and fins.

## Supplementary Material

obac035_Supplemental_files

## Data Availability

The data underlying this article are available in its online supplementary material.
