# Supplementary material for: Extraoral Taste Buds on the Paired Fins of Damselfishes
Source: Integr Org Biol. 2022 Aug 11;4(1):obac035. doi: 10.1093/iob/obac035 (PMC9428928; doi:10.1093/iob/obac035)
Supplement: obac035_Supplemental_files [file obac035_supplemental_files.zip › Ephys_Taste_Response_Data.pdf]

Fish ID	Experiment ID #	Stimulus/Control	Trial#	Afferent ID #	Stimulus Duration (s)	Total Spike #	Burst Spike#	Start Burst(s)	End Burst(s)	Burst Duration (s)	Mean Burst ISI (s)	Burst Spike Rate (spikes/s)
201117xxx	1	Stimulus	201117015	1	3	77	70	6.201	12.760	6.559	0.095	10.673
201117xxx	1	Stimulus	201117015	2	3	40	36	1.931	14.256	12.325	0.352	2.921
201117xxx	1	Stimulus	201117015	3	3	31	30	7.972	12.571	4.600	0.159	6.522
201117xxx	1	Control	201117016	1	3							
201117xxx	1	Control	201117016	2	3	21	15	2.748	13.059	10.311	0.807	1.217
201117xxx	1	Control	201117016	3	3							
201117xxx	1	Stimulus	201117017	1	3	57	56	6.327	13.777	7.450	0.135	7.517
201117xxx	1	Stimulus	201117017	2	3	48	41	3.018	17.956	14.937	0.373	2.745
201117xxx	1	Stimulus	201117017	3	3	27	27	8.337	14.252	5.915	0.228	4.564
201117xxx	2	Stimulus	201117019	1	3	40	40	2.059	17.123	15.064	0.386	2.655
201117xxx	2	Stimulus	201117019	3	3	124	122	2.638	14.720	12.082	0.100	10.097
201117xxx	2	Stimulus	201117019	4	3	20	20	5.387	12.060	6.673	0.351	2.997
201117xxx	2	Stimulus	201117019	5	3	23	23	6.211	11.812	5.601	0.255	4.106
201117xxx	2	Control	201117022	1	3	15	15	2.126	12.775	10.649	0.761	0.996
201117xxx	2	Control	201117022	3	3							
201117xxx	2	Control	201117022	4	3	27	12	6.227	11.519	5.291	0.536	1.798
201117xxx	2	Control	201117022	5	3							
201117xxx	2	Stimulus	201117024	1	3	21	21	2.534	13.277	10.743	0.537	1.955
201117xxx	2	Stimulus	201117024	3	3	120	118	3.330	13.705	10.375	0.089	11.373
201117xxx	2	Stimulus	201117024	4	3	33	32	3.774	11.817	8.043	0.259	3.979
201117xxx	2	Stimulus	201117024	5	3	24	24	6.662	12.892	6.230	0.271	3.853
201117xxx	3	Stimulus	201117025	1	3	114	111	2.109	19.726	17.618	0.160	6.300
201117xxx	3	Stimulus	201117025	2	3	65	64	2.071	11.697	9.627	0.153	6.648
201117xxx	3	Control	201117026	1	3	20	18	3.159	19.706	16.547	0.992	1.022
201117xxx	3	Control	201117026	2	3							
201117xxx	3	Stimulus	201117027	1	3	104	103	1.592	29.626	28.035	0.275	3.674
201117xxx	3	Stimulus	201117027	2	3	29	29	2.045	11.412	9.367	0.335	3.096
201215xxx	4	Stimulus	201215011	1	5	14	14	7.937	12.408	4.471	0.344	3.131
201215xxx	4	Control	201215012	1	5							
201215xxx	4	Stimulus	201215016	1	5	46	45	6.673	12.165	5.492	0.125	8.193
201215xxx	5	Stimulus	201215025	1	5	15	15	8.887	17.062	8.175	0.584	1.835
201215xxx	5	Stimulus	201215025	2	5	17	17	11.226	14.859	3.633	0.227	4.679
201215xxx	5	Control	201215026	1	5							
201215xxx	5	Control	201215026	2	5							
201215xxx	5	Stimulus	201215028	1	5	18	18	7.946	11.613	3.667	0.216	4.909
201215xxx	5	Stimulus	201215028	2	5	17	17	10.524	13.697	3.173	0.198	5.358
201215xxx	6	Stimulus	201215032	1	5	17	17	5.675	17.484	11.809	0.738	1.440
201215xxx	6	Stimulus	201215032	2	5	12	12	7.679	9.944	2.266	0.206	5.297
201215xxx	6	Control	201215033	1	5							
201215xxx	6	Control	201215033	2	5							
201215xxx	6	Stimulus	201215034	1	5	39	38	5.592	9.763	4.171	0.113	9.110
201215xxx	6	Stimulus	201215034	2	5	15	15	6.218	8.957	2.739	0.196	5.476
201215xxx	7	Stimulus	201215035	1	5	46	43	5.612	10.548	4.936	0.118	8.712
201215xxx	7	Control	201215036	1	5							
201215xxx	7	Stimulus	201215037	1	5	26	26	5.303	10.318	5.015	0.201	5.185
201218xxx	8	Stimulus	201218011	1	5	22	22	8.480	12.887	4.407	0.210	4.992
201218xxx	8	Control	201218012	1	5							
201218xxx	8	Stimulus	201218014	1	5	70	70	5.771	16.023	10.253	0.149	6.828
201218xxx	9	Stimulus	201218022	1	5	48	47	3.060	8.337	5.277	0.115	8.906
201218xxx	9	Stimulus	201218022	2	5	21	21	3.179	7.493	4.314	0.216	4.868
201218xxx	9	Control	201218023	1	5							
201218xxx	9	Control	201218023	2	5	5	1	6.532	6.532	0.000		0.232
201218xxx	9	Stimulus	201218026	1	5	35	34	2.550	8.709	6.160	0.187	5.520
201218xxx	9	Stimulus	201218026	2	5	26	24	2.494	7.186	4.693	0.204	5.114
201218xxx	10	Stimulus	201218032	1	5	23	23	6.083	10.035	3.952	0.180	5.820
201218xxx	10	Control	201218033	1	5							
201218xxx	10	Stimulus	201218035	1	5	36	36	4.584	7.137	2.553	0.073	14.102
