# Supplementary material for: Extraoral Taste Buds on the Paired Fins of Damselfishes
Source: Integr Org Biol. 2022 Aug 11;4(1):obac035. doi: 10.1093/iob/obac035 (PMC9428928; doi:10.1093/iob/obac035)
Supplement: obac035_Supplemental_files [file obac035_supplemental_files.zip › Taste_bud_density_data.pdf]

Taste Bud Density (cells/mm2)

	LE	FR #6	FR #9	FR #11	FR #15	TE
Chromis viridis_01_Pectoral_L						
Distal Tips	240.91	68.72	99.48	80.35	108.96	148.30
	84.47	32.63	35.26	25.04	53.81	139.82
	87.35	7.13	10.58	22.93	33.69	141.52
	107.90	0.00	4.28	10.19	14.78	32.53
Proximal Base	102.23	8.71	16.70	20.81	22.53	24.86
Chromis viridis_01_Pectoral_R	74.13	69.02	77.80	94.64	51.27	162.81
	57.95	17.78	46.18	38.23	40.43	171.05
	80.84	5.72	19.36	18.23	30.62	116.87
	40.94	0.00	0.00	0.00	15.16	58.87
	57.20	0.00	4.80	6.02	0.00	35.05
Chromis viridis_02_Pectoral_L	35.50	48.35	22.92	32.88	99.23	67.60
	19.55	10.27	9.14	27.21	35.39	61.58
	31.21	27.46	5.58	19.58	25.75	73.54
	38.50	8.75	0.00	8.08	3.99	25.14
	86.25	4.06	11.91	10.67	0.00	0.00
Chromis viridis_02_Pectoral_R	17.73	52.83	49.91	54.27	86.50	132.82
	1.84	20.89	24.17	20.79	48.17	119.99
	24.05	4.81	13.03	15.43	22.80	82.59
	33.53	2.11	1.94	0.00	0.00	21.65
	49.80	0.00	7.60	0.00	0.00	0.00
Chromis viridis_03_Pectoral_L	62.52	52.71	70.31	58.75	56.83	31.59
	59.73	38.14	52.44	56.38	23.20	176.98
	27.18	21.56	12.61	26.77	30.12	64.82
	40.11	12.37	12.42	16.69	23.45	80.12
	56.35	11.06	0.00	0.00	6.51	78.70
Chromis viridis_03_Pectoral_R	56.47	67.80	38.55	63.83	62.05	84.72
	69.18	37.08	44.39	48.61	30.92	149.29
	24.07	19.59	12.30	13.47	32.01	47.53
	30.48	0.00	2.59	2.90	17.07	55.86
	12.42	0.00	0.00	0.00	30.55	67.64
	81.21	59.90	59.83	64.12	77.47	104.64
	48.79	26.13	35.26	36.04	38.65	136.45
Average	45.79	14.38	12.24	19.40	29.16	87.81
	48.58	3.87	3.54	6.31	12.41	45.70
	60.71	3.97	6.83	6.25	9.93	34.38
	80.79	9.58	27.99	21.43	24.07	51.36
	31.47	11.45	16.14	14.20	11.25	42.17
Std Dev	29.86	9.69	4.44	4.88	4.09	34.96
	29.34	5.37	4.65	6.59	8.74	22.96
	31.17	4.90	6.65	8.35	13.35	33.24

Taste Bud Density (cells/mm2)

	LE	FR #4	TE
Chromis viridis_01_Pelvic_L			
**Distal Tips**	150.83	111.39	81.92
	117.49	60.60	53.35
	58.59	49.99	39.32
	49.53	32.00	33.48
**Proximal Base**	50.34	17.87	15.38
Chromis viridis_01_Pelvic_R	111.62	112.66	108.66
	59.48	66.54	98.92
	43.56	57.05	58.97
	14.17	12.99	0.00
	6.00	7.40	7.43
Chromis viridis_02_Pelvic_L	198.27	78.46	36.15
	83.59	26.67	17.72
	38.55	10.95	10.53
	5.76	10.17	6.74
	8.91	15.50	0.00
Chromis viridis_02_Pelvic_R	232.86	65.05	85.15
	80.35	31.14	26.10
	54.60	25.95	15.62
	41.56	14.89	10.82
	27.73	9.91	4.87
Chromis viridis_03_Pelvic_L	211.87	32.66	27.59
	115.31	29.52	14.16
	42.17	22.19	14.70
	41.36	22.57	11.13
	49.32	12.22	0.00
Chromis viridis_03_Pelvic_R	245.53	63.74	NaN
	82.74	29.41	NaN
	53.44	28.23	NaN
	49.42	20.86	NaN
	14.33	7.97	NaN
	191.83	77.33	67.89
	89.83	40.65	42.05
**Average**	48.48	32.39	27.83
	33.63	18.91	12.43
	26.11	11.81	5.53
	51.23	30.79	34.60
	22.42	17.91	35.31
**Std Dev**	8.09	17.55	20.75
	18.87	7.95	12.59
	19.84	4.20	6.37

Taste Bud Density (cells/mm2)

	LE	FR #5	FR #8	FR #10	FR #13	TE
Pomacentrus amboinensis_01_Pectoral_L						
Distal Tips	77.20	29.05	16.11	9.86	25.79	125.28
	64.26	8.81	4.64	3.71	16.40	57.04
	87.77	6.32	3.69	4.18	11.81	78.75
	53.69	7.18	2.41	2.61	0.00	63.45
Proximal Base	43.34	0.00	0.00	0.00	9.23	75.43
Pomacentrus amboinensis_01_Pectoral_R	68.80	14.22	17.20	37.27	34.37	82.88
	60.42	10.01	4.49	12.49	8.19	46.68
	68.86	2.05	1.92	2.35	0.00	43.18
	53.71	0.00	0.00	0.00	0.00	37.41
	13.54	0.00	0.00	8.18	17.16	77.12
Pomacentrus amboinensis_02_Pectoral_L	62.61	18.74	NaN	NaN	18.31	105.41
	48.92	10.71	NaN	NaN	8.66	92.12
	34.76	6.79	NaN	NaN	9.61	112.66
	25.79	0.00	NaN	NaN	0.00	31.28
	27.81	6.69	NaN	NaN	0.00	36.93
Pomacentrus amboinensis_02_Pectoral_R	56.45	23.32	13.26	17.73	17.05	NaN
	32.42	15.94	8.26	17.60	19.20	NaN
	50.50	7.28	12.37	11.35	14.42	NaN
	49.92	0.00	0.00	0.00	0.00	NaN
	26.41	5.37	1.99	0.00	0.00	NaN
Pomacentrus amboinensis_03_Pectoral_L	120.47	29.61	26.12	22.97	21.18	98.39
	101.27	19.43	20.26	18.56	22.76	91.09
	56.08	5.35	13.68	11.03	13.64	55.99
	42.79	1.74	2.11	2.70	8.15	43.49
	30.11	0.00	11.83	3.44	0.00	60.89
Pomacentrus amboinensis_03_Pectoral_R	69.11	31.33	21.45	15.81	44.70	135.84
	21.27	20.87	17.70	14.04	18.22	36.61
	47.94	11.91	12.07	12.93	14.56	60.01
	68.34	1.82	9.89	2.53	0.00	47.59
	33.52	2.01	0.00	0.00	4.21	79.71
Average	75.77	24.38	18.83	20.73	26.90	109.56
	54.76	14.29	11.07	13.28	15.57	64.71
	57.65	6.61	8.75	8.37	10.67	70.12
	49.04	1.79	2.88	1.57	1.36	44.64
	29.12	2.34	2.76	2.33	5.10	66.02
Std Dev	22.98	6.83	5.03	10.37	10.75	21.16
	28.07	5.17	7.43	5.90	5.92	25.60
	18.46	3.20	5.49	4.76	5.56	26.98
	14.12	2.78	4.08	1.43	3.33	12.19
	9.74	2.99	5.14	3.60	6.95	17.83

Taste Bud Density (cells/mm2)

	LE	FR #4	TE
Pomacentrus amboinensis_01_Pelvic_L			
Distal Tips	91.32	11.19	NaN
	23.25	2.32	NaN
	18.30	3.58	NaN
	6.51	0.00	NaN
Proximal Base	4.28	0.00	NaN
Pomacentrus amboinensis_01_Pelvic_R	126.55	14.55	28.05
	40.43	4.45	0.00
	27.64	0.00	0.00
	14.51	0.00	0.00
	16.01	0.00	0.00
Pomacentrus amboinensis_02_Pelvic_L	80.11	21.49	20.57
	63.16	4.08	0.00
	22.50	0.00	0.00
	7.43	2.26	0.00
	3.93	0.00	0.00
Pomacentrus amboinensis_02_Pelvic_R	58.39	10.98	NaN
	59.61	2.59	NaN
	29.96	0.00	NaN
	15.01	0.00	NaN
	19.15	0.00	NaN
Pomacentrus amboinensis_03_Pelvic_L	126.83	18.42	2.58
	52.50	8.26	0.00
	20.58	4.50	3.61
	9.05	3.17	2.17
	9.86	0.00	0.00
Pomacentrus amboinensis_03_Pelvic_R	119.59	3.91	0.00
	52.36	6.03	1.76
	17.12	3.40	0.00
	7.54	0.00	2.30
	8.76	0.00	0.00
	100.46	13.42	12.80
	48.55	4.62	0.44
Average	22.68	1.91	0.90
	10.01	0.90	1.12
	10.33	0.00	0.00
	28.32	6.21	13.68
	14.64	2.24	0.88
Std Dev	5.14	2.13	1.81
	3.77	1.43	1.29
	6.17	0.00	0.00

Taste Bud Density (cells/mm2)

	LE	FR #5	FR #8	FR #10	FR #14	TE
Pomacentrus coelestis_01_Pectoral_L						
Distal Tips	46.13	31.91	22.13	NaN	NaN	81.33
	49.11	15.20	12.87	NaN	NaN	114.45
	99.18	7.64	5.14	NaN	NaN	76.66
	66.66	0.00	0.00	NaN	NaN	71.97
Proximal Base	10.44	0.00	2.03	NaN	NaN	19.31
Pomacentrus coelestis_01_Pectoral_R	63.81	NaN	31.36	NaN	NaN	64.87
	29.74	NaN	18.08	NaN	NaN	72.91
	53.94	NaN	2.07	NaN	NaN	74.71
	28.73	NaN	0.00	NaN	NaN	51.14
	20.37	NaN	0.00	NaN	NaN	0.00
Pomacentrus coelestis_02_Pectoral_L	15.34	20.87	29.38	14.72	35.15	86.94
	36.22	22.33	16.83	14.66	12.50	111.09
	46.81	7.20	6.78	7.67	15.70	83.18
	68.00	1.19	0.00	1.61	0.00	51.88
	32.46	2.66	0.00	0.00	0.00	59.90
Pomacentrus coelestis_02_Pectoral_R	71.53	25.08	12.65	14.21	27.59	153.74
	48.80	17.86	14.84	16.05	19.22	173.85
	113.96	6.97	12.43	10.06	7.31	127.42
	91.78	0.00	1.34	0.00	0.00	90.54
	99.20	0.00	0.00	1.43	0.00	43.98
Pomacentrus coelestis_03_Pectoral_L	115.32	34.08	28.42	31.91	36.69	78.25
	28.36	18.74	18.62	20.24	14.75	97.03
	29.16	9.89	6.50	9.88	12.04	63.39
	32.67	3.11	3.34	2.76	4.64	44.68
	29.82	0.00	0.00	0.00	0.00	58.19
Pomacentrus coelestis_03_Pectoral_R	78.03	33.46	16.65	26.21	23.35	82.22
	37.49	20.71	16.04	11.14	16.10	83.51
	27.66	8.84	9.09	6.24	10.78	69.51
	23.32	2.83	1.01	0.00	4.39	59.47
	18.30	0.00	4.41	0.00	0.00	73.33
Average	65.03	29.08	23.43	21.76	30.70	91.23
	38.29	18.97	16.21	15.52	15.64	108.81
	61.79	8.11	7.00	8.46	11.46	82.48
	51.86	1.43	0.95	1.09	2.26	61.61
	35.10	0.53	1.07	0.36	0.00	42.45
Std Dev	33.37	5.82	7.57	8.74	6.31	31.52
	8.99	2.73	2.13	3.76	2.81	35.60
	36.43	1.23	3.52	1.84	3.46	23.01
	27.54	1.49	1.31	1.35	2.61	16.97
	32.41	1.19	1.83	0.72	0.00	27.73

Taste Bud Density (cells/mm2)

	LE	FR #4	TE
Pomacentrus coelestis_01_Pelvic_L			
Distal Tips	146.12	9.18	0.00
	80.46	16.76	8.67
	32.48	33.88	0.00
	21.46	10.61	3.74
Proximal Base	20.30	0.00	6.85
Pomacentrus coelestis_01_Pelvic_R	126.02	20.36	4.94
	66.70	8.29	0.00
	39.61	8.07	0.00
	5.57	14.01	0.00
	0.94	5.31	2.84
Pomacentrus coelestis_02_Pelvic_L	106.57	14.07	0.00
	42.08	3.31	0.00
	32.55	0.00	2.81
	9.87	0.00	0.00
	10.33	0.00	0.00
Pomacentrus coelestis_02_Pelvic_R	96.85	15.10	0.00
	31.68	1.00	0.00
	31.61	0.00	0.00
	10.64	0.00	0.00
	6.60	0.00	0.00
Pomacentrus coelestis_03_Pelvic_L	125.06	24.42	12.12
	45.46	5.14	9.03
	23.04	3.66	11.41
	19.27	4.31	6.07
	5.12	0.00	0.00
Pomacentrus coelestis_03_Pelvic_R	95.43	17.62	6.77
	30.71	6.58	5.31
	21.00	6.12	10.64
	18.21	2.64	0.00
	11.25	0.00	0.00
	116.01	16.79	3.97
	49.52	6.85	3.83
Average	30.05	8.62	4.14
	14.17	5.26	1.63
	9.09	0.88	1.62
	19.84	5.29	4.95
	19.98	5.48	4.40
Std Dev	6.89	12.79	5.44
	6.33	5.80	2.64
	6.64	2.17	2.80
